# Right Site, Wrong Route ― Cannulating the Left Internal Jugular Vein

**DOI:** 10.7759/cureus.2044

**Published:** 2018-01-09

**Authors:** Peter Paik, Sanjay K Arukala, Anupam A Sule

**Affiliations:** 1 Internal Medicine, St. Joseph Mercy Oakland Hospital

**Keywords:** congenital anomaly, vascular malformation, vascular access, left superior vena cava

## Abstract

Central venous catheters are placed in approximately five million patients annually in the US. The preferred site of insertion is one with fewer risks and easier access. Although the right internal jugular vein is preferred, on occasion, the left internal jugular may have to be accessed. A patient was admitted for septic shock, cerebrovascular accident, and non-ST-segment elevation myocardial infarction. A central venous line was needed for antibiotic and vasopressor administration. Due to trauma from a fall to the right side and previously failed catheterization attempts at the left subclavian and femoral veins, the left internal jugular vein was accessed. On chest radiography for confirmation, the left internal jugular central venous catheter was seen projecting down the left paraspinal region. It did not take the expected course across the midline toward the right and into the superior vena cava (SVC). A review of a computed tomography (CT) scan of the chest with contrast done on a prior admission revealed a duplicated SVC on the left side that had not been reported in the original CT scan interpretation. A left-sided SVC is present in approximately 0.3% to 0.5% of the population, with 90% of these draining into the coronary sinus. During placements of central venous lines and pacemakers, irritation of the coronary sinus may result in hypotension, arrhythmia, myocardial ischemia, or cardiac arrest. A widened mediastinum can be an indication of a duplicated SVC. When attempting a left internal jugular vein central venous catheter placement, it is important to be aware of venous anomalies in order to prevent complications.

## Introduction

A left-sided superior vena cava (SVC) is present in approximately 0.3% to 0.5% of the population, with 90% of these draining into the coronary sinus [1-4]. A widened mediastinum can be an indication of a duplicated SVC [3]. During placements of central venous lines and pacemakers, irritation of the coronary sinus may result in hypotension, arrhythmia, myocardial ischemia, or cardiac arrest. Hence, when attempting left internal jugular vein central venous catheter placement, it is important to be aware of venous anomalies to prevent these complications [1-2].

## Case presentation

A 58-year-old man with a history of alcohol abuse presented to the hospital after being found unresponsive. Per the patient’s ex-wife, the patient fell and struck his head the prior night and was found lying on the couch. On the first examination by the emergency medical services (EMS), he was unresponsive, with a left-sided facial droop. In the emergency department, the patient was intubated. His blood pressure was 95/61 mmHg, the heart rate was 94 beats per minute, and the temperature was 99.7 degrees F. A CT scan of the head and cervical spine (C-spine) ruled out any acute process from trauma. Significant laboratory values included a bicarbonate of 5.4 mMol/L, an anion gap of 23 mEq/L, glucose of 419 mg/dL, and a pH of 7.10, which were suggestive of diabetic ketoacidosis. The patient was started on an insulin infusion and transferred to the intensive care unit.

**Figure 1:**
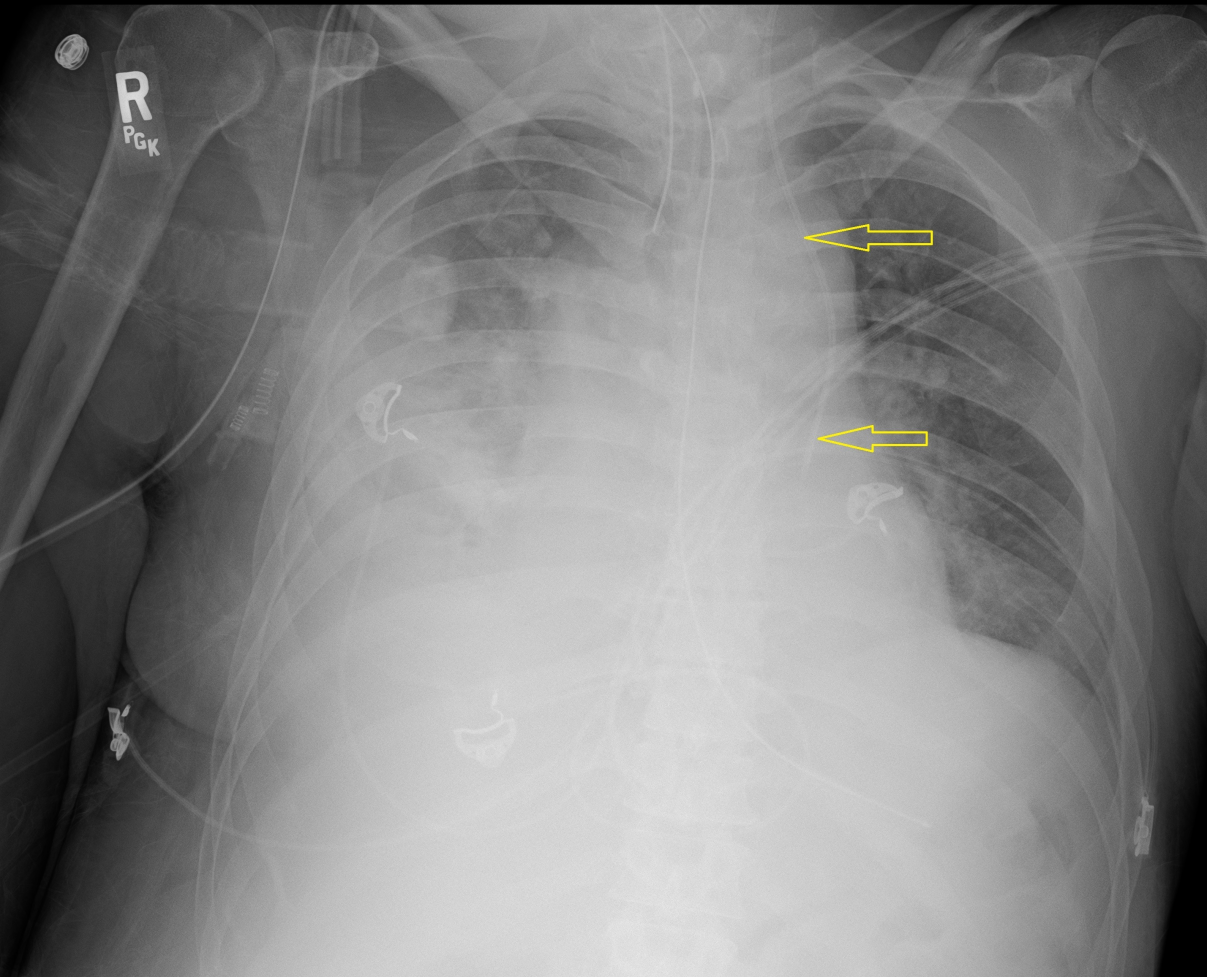
Chest x-ray confirming the placement of the central venous access line. The line (marked by yellow arrows) does not take the expected course toward the right side of the heart.

He was diagnosed with septic shock secondary to healthcare-associated pneumonia, a cerebrovascular accident due to ischemic infarct in the left frontal lobe, and non-ST-segment elevation myocardial infarction. A central venous line was needed for antibiotic and vasopressor administration. Due to superficial abrasions from the fall to the right side of his neck, the left internal jugular vein was cannulated. On chest radiography (Figure 1) for confirmation, the left internal jugular central venous catheter was seen projecting down the left paraspinal region. It did not take the expected course across the midline toward the right and into the SVC.

**Figure 2:**
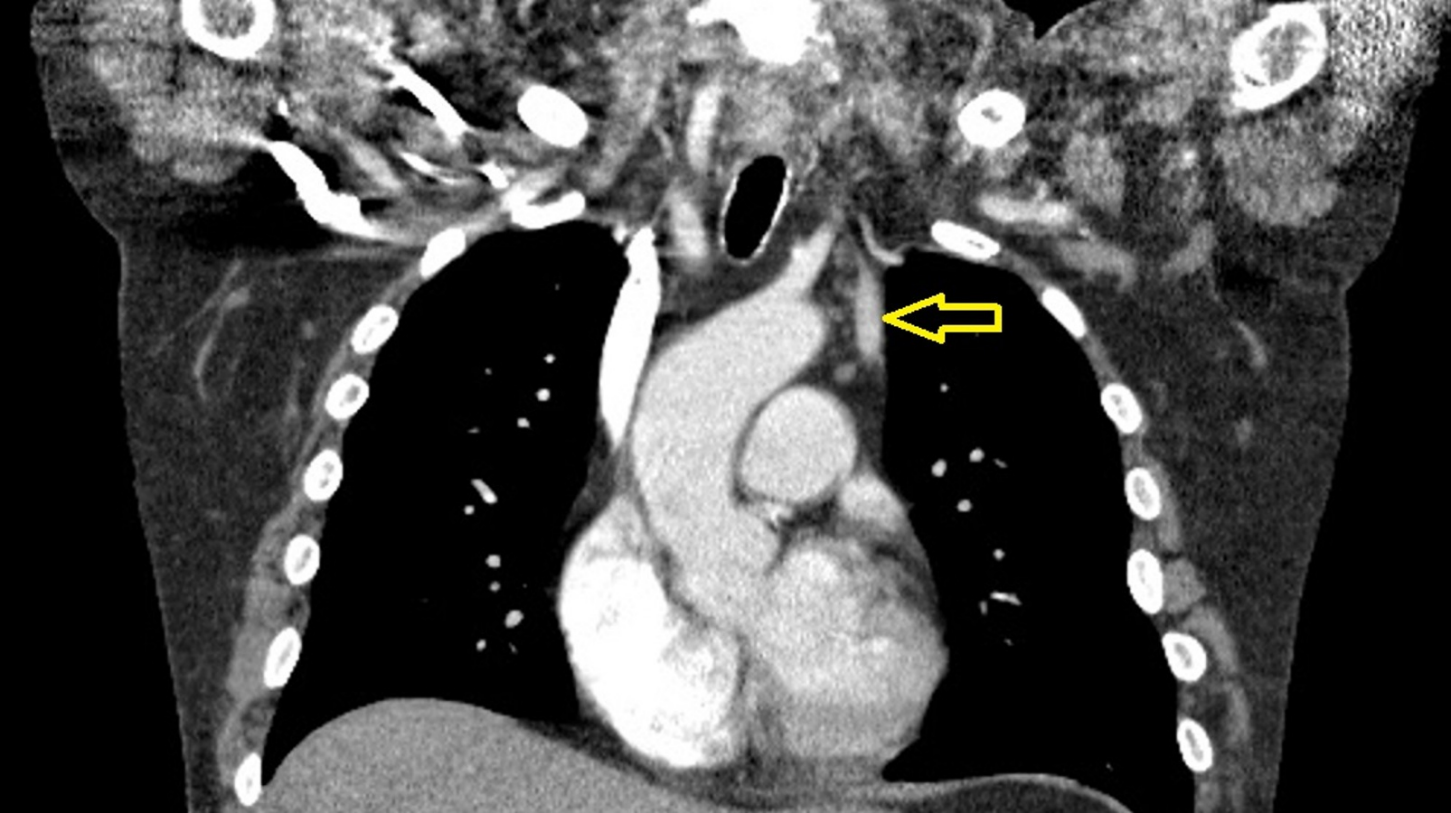
Chest computed tomography (CT) scan (coronal view) with contrast performed on a prior admission confirming the presence of a duplicated superior vena cava (SVC) (marked by yellow arrow) draining into the coronary sinus.

**Figure 3:**
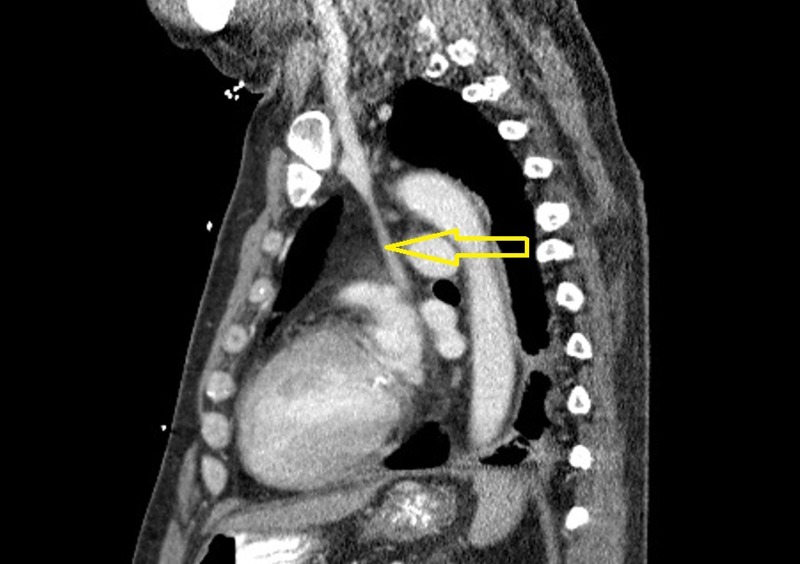
Chest computerized tomography (CT) scan (sagittal view) with contrast performed on a prior admission confirming the presence of a duplicated superior vena cava (SVC) (marked by yellow arrow) draining into the coronary sinus.

Given this imagining, a CT chest with contrast (Figure 2 and Figure 3) done on a prior admission was reviewed, which revealed a duplicated SVC on the left side that had not been reported in the original CT scan interpretation.

**Figure 4:**
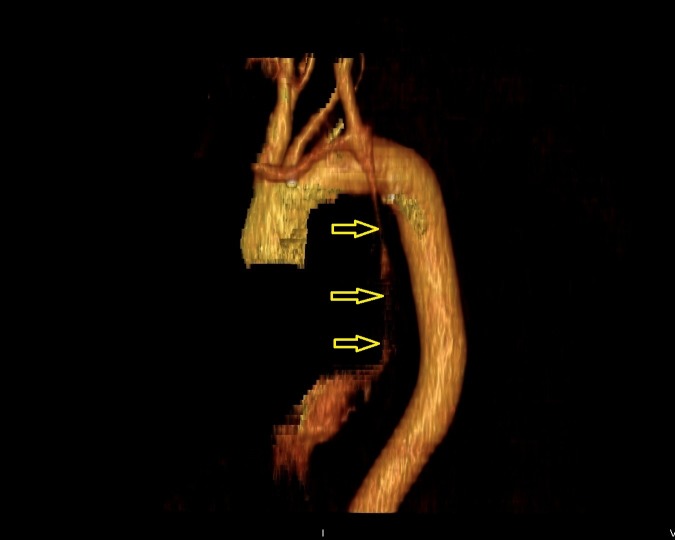
Three-dimensional (3D) reconstruction of the patient’s vasculature, highlighting the presence of the duplicated superior vena cava (SVC) (marked by yellow arrows) draining into the coronary sinus.

Three-dimensional (3-D) reconstruction (Figure 4) confirmed the presence of the duplicated SVC.

The patient remained stable following the placement of the central line, and he was educated on the significance of his anatomical anomaly. Other congenital cardiac anomalies were not detected on a transthoracic echocardiogram performed during hospitalization.

## Discussion

Venocaval anatomical variations are relatively common and are important to recognize. A duplicated SVC is an incidental finding, as it rarely causes symptoms. In the case of this patient, a duplicated superior vena cava was suspected after a chest radiograph was used to confirm the placement of a central venous access line. The diagnosis was subsequently confirmed via a CT scan. A left-sided superior vena cava is present in approximately 0.3% to 0.5% of the population [1]. In patients with congenital heart diseases, the number is significantly higher at 4.3% to 11% [1]. A widened mediastinum can be an indication of a duplicated superior vena cava [1-4]. During embryological development, the left common cardinal and the left precardinal veins normally regress completely, leaving only the right anterior cardinal vein to continue as the superior vena cava [3]. The complicated development of the SVC allows for many variations to develop. There are four main variants recorded due to this developmental stage: single right-sided SVC, double SVC with left and right draining into the right atrium, double SVC with each draining into each respective atrium, and a single persistent left SVC that empties in the left atrium. Over 90% of left-sided SVCs drain into the right atrium via the coronary sinus, as in the case of our patient. The remaining 10% drain directly into the left atrium, potentially causing hemodynamic instabilities [5]. Left-sided SVCs that drain into the left atrium are more problematic, as the heart develops a right-to-left shunt [3]. Patients are typically asymptomatic as well but would have a greater propensity to develop right-sided heart failure because of this shunt. Venous return going to the left atrium puts the patient at significant risk of developing embolisms within the arterial system [5]. When a patient is known to have a duplicated SVC on the left and central venous access is being obtained, it is important to recognize their specific pattern of cardiac venous return prior to utilizing the catheter.

## Conclusions

When attempting a left internal jugular vein central venous catheter placement, it is important to be aware of venous anomalies especially in the presence of a widened mediastinum on the chest X-ray. During these placements, irritation of the coronary sinus could cause severe hypotension, arrhythmia, myocardial ischemia, or cardiac arrest. Observation of the cardiac rhythm during the advancement of the guide wire may provide an early indication of coronary sinus irritation in patients with anatomical abnormalities of the SVC.
